# Supplementary material for: Systems-Scale Structural Modeling Reveals the Germline Architecture of Immunodominance
Source: bioRxiv. 2026 Jun 4:2026.06.02.729678. Preprint. [Version 1] doi: 10.64898/2026.06.02.729678 (PMC13252370; doi:10.64898/2026.06.02.729678)
Supplement: Supplement 1 — Figure S1. Antibodies to immunodominant wild-type and BA.5 RBD epitopes exhibit biased V gene segment usage, suggesting germline-encoded recognition. (A) Heavy-light V gene segment usage of antibodies that recognize the wild-type RBD, NTD, and S2 subdomains of SARS-CoV-2 spike, as in Figure 1A, with labels. (B) BA.5 RBD DMS dataset, clustered and visualized as in Figure 1B. (C and D) Heavy-light V gene segment usage of antibodies in each (C) wild-type RBD or (D) BA.5 DMS Epitope Cluster, as in Figure 1C, with labels. Figure S2. There is a strong association between gene segment usage and epitope specificity. (A and B) Comparison of the epitopes of antibodies with highly enriched heavy-light V gene segment pairs. To visualize and cluster antibodies based on their wild-type RBD DMS epitope maps, we applied t-distributed stochastic neighbor embedding (t-SNE) to reduce the feature space to two dimensions, then performed k-means clustering. This clustering method is the same as was used for Figure 1B. Points representing antibodies with particular overrepresented V gene segment pairs are shown in color, while points representing all other antibodies are in light grey. (C) Average wild-type RBD DMS data for unique-lineage antibodies that share overrepresented heavy-light V gene segment pairs. A DMS escape score of ≥ 0.3 was applied as a threshold to define critical RBD epitope residues for each antibody (see Methods). Then the DMS data, now converted to 0 or 1 binary values, were averaged for all antibodies with the indicated heavy-light V gene segment pairs. To limit figure size, only heavy-light V gene segment pairs enriched over their usage in the naïve antibody repertoire with FDR < 1.0 x 10^−7^ were plotted. Rows in the heatmap were clustered using hierarchical clustering. Rows representing antibodies with overrepresented V gene segment pairs mentioned in (A) and (B) are shown in color and labeled with grey circled numbers at left that correspond with grey circled numb [file media-1.pdf]

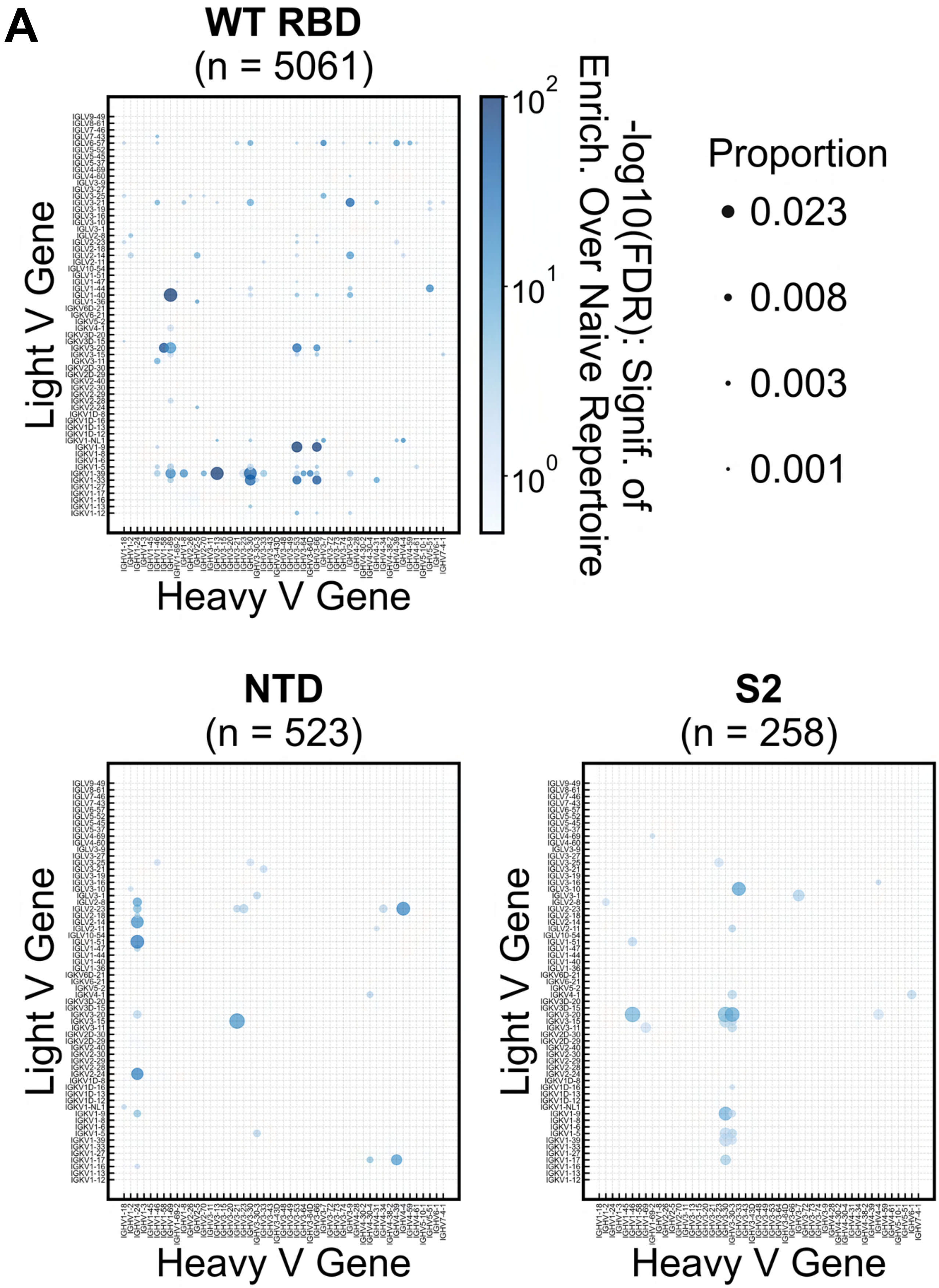
A
WT RBD
(n = 5061)
NTD
(n = 523)
S2
(n = 258)
Light V Gene
Heavy V Gene
-log10(FDR): Signif. of Enrich. Over Naive Repertoire
10^2
10^1
10^0
Proportion
0.023
0.008
0.003
0.001

Figure S1

B

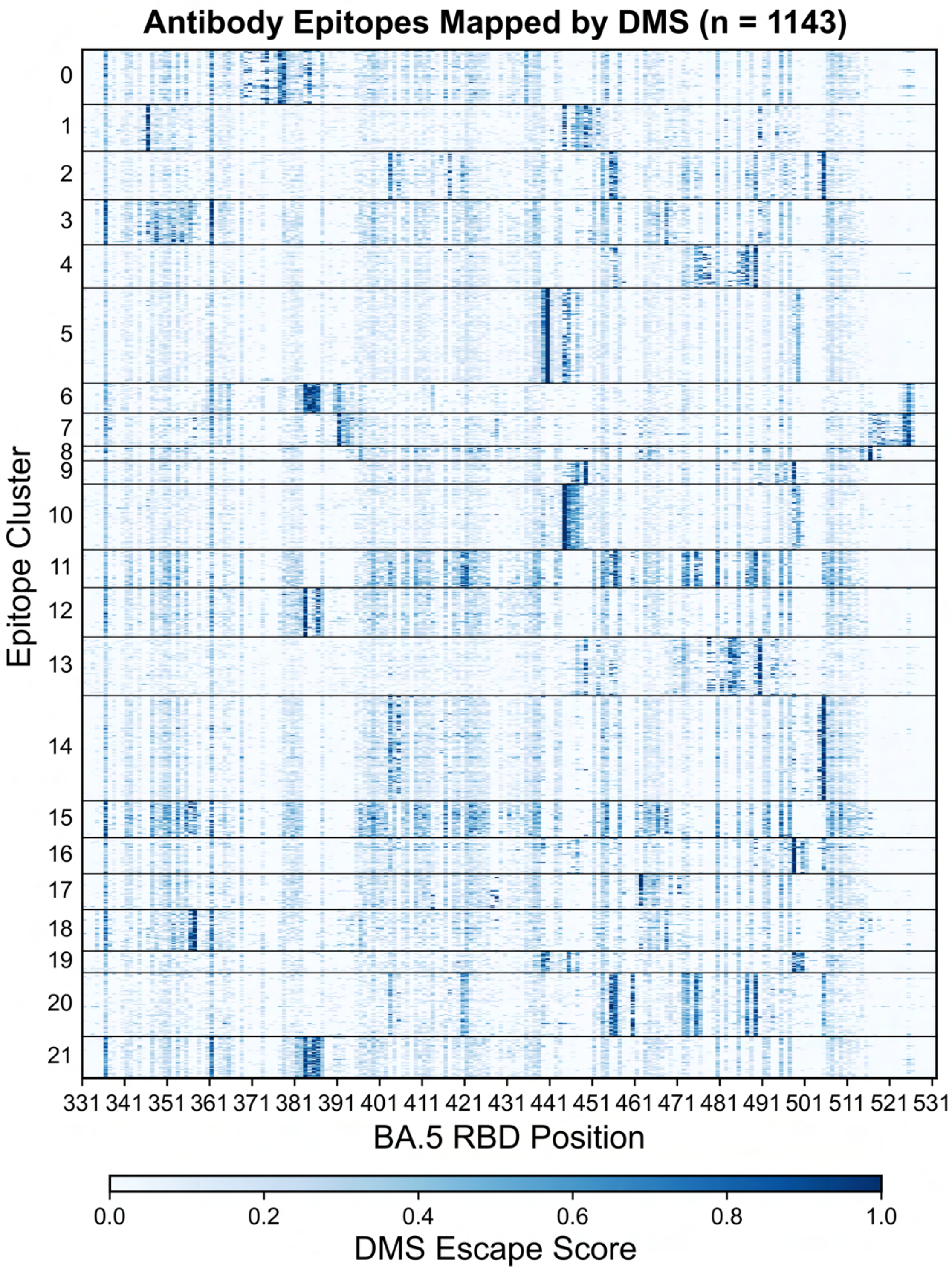

Figure S1 continued

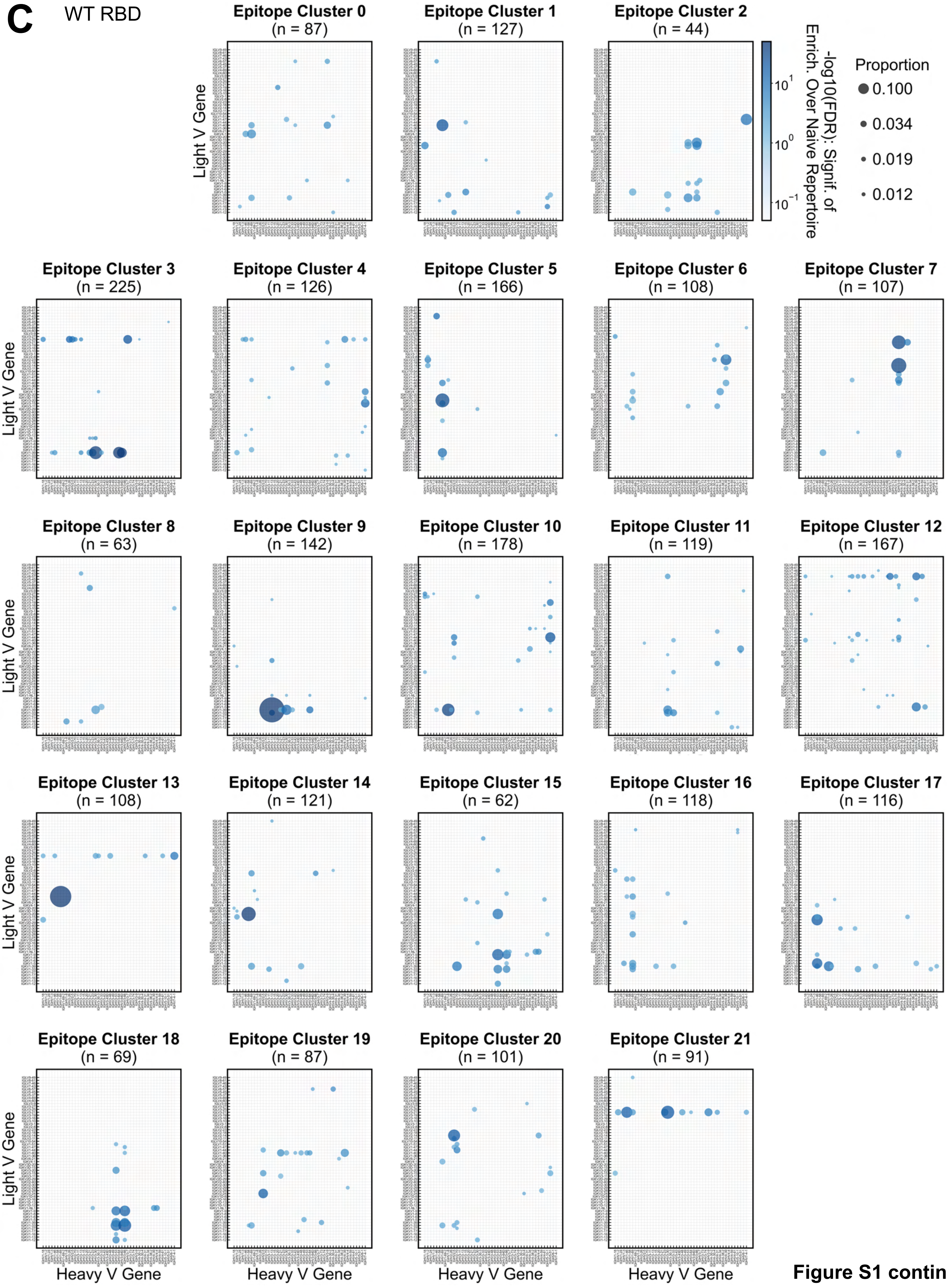

Figure S1 continued

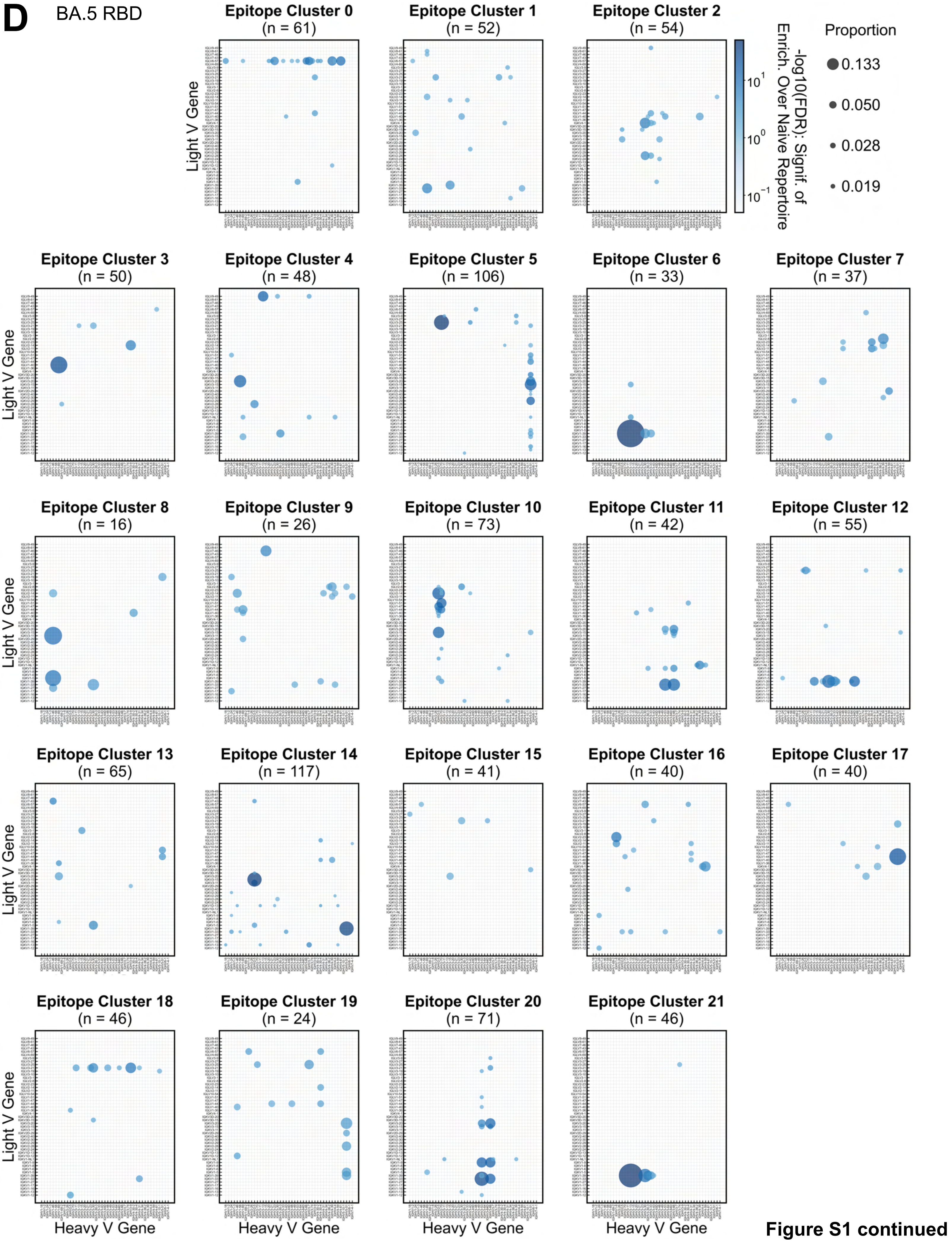

Figure S1 continued

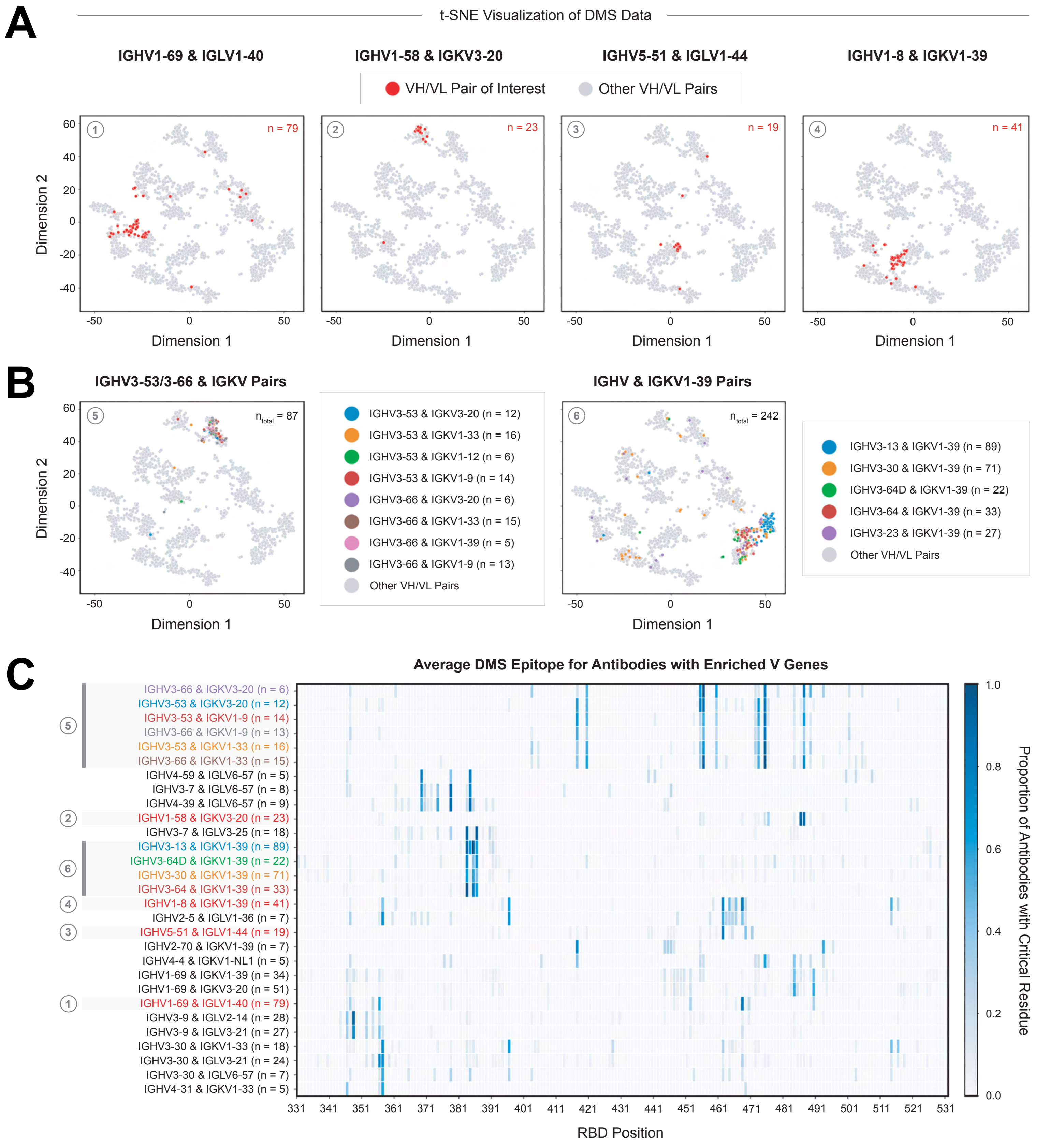
A
t-SNE Visualization of DMS Data
IGHV1-69 & IGLV1-40
IGHV1-58 & IGKV3-20
IGHV5-51 & IGLV1-44
IGHV1-8 & IGKV1-39
VH/VL Pair of Interest
Other VH/VL Pairs
n = 79
n = 23
n = 19
n = 41
Dimension 1
Dimension 2
B
IGHV3-53/3-66 & IGKV Pairs
IGHV & IGKV1-39 Pairs
n_{total} = 87
n_{total} = 242
IGHV3-53 & IGKV3-20 (n = 12)
IGHV3-53 & IGKV1-33 (n = 16)
IGHV3-53 & IGKV1-12 (n = 6)
IGHV3-53 & IGKV1-9 (n = 14)
IGHV3-66 & IGKV3-20 (n = 6)
IGHV3-66 & IGKV1-33 (n = 15)
IGHV3-66 & IGKV1-39 (n = 5)
IGHV3-66 & IGKV1-9 (n = 13)
Other VH/VL Pairs
IGHV3-13 & IGKV1-39 (n = 89)
IGHV3-30 & IGKV1-39 (n = 71)
IGHV3-64D & IGKV1-39 (n = 22)
IGHV3-64 & IGKV1-39 (n = 33)
IGHV3-23 & IGKV1-39 (n = 27)
Other VH/VL Pairs
C
Average DMS Epitope for Antibodies with Enriched V Genes
IGHV3-66 & IGKV3-20 (n = 6)
IGHV3-53 & IGKV3-20 (n = 12)
IGHV3-53 & IGKV1-9 (n = 14)
IGHV3-66 & IGKV1-9 (n = 13)
IGHV3-53 & IGKV1-33 (n = 16)
IGHV3-66 & IGKV1-33 (n = 15)
IGHV4-59 & IGLV6-57 (n = 5)
IGHV3-7 & IGLV6-57 (n = 8)
IGHV4-39 & IGLV6-57 (n = 9)
IGHV1-58 & IGKV3-20 (n = 23)
IGHV3-7 & IGLV3-25 (n = 18)
IGHV3-13 & IGKV1-39 (n = 89)
IGHV3-64D & IGKV1-39 (n = 22)
IGHV3-30 & IGKV1-39 (n = 71)
IGHV3-64 & IGKV1-39 (n = 33)
IGHV1-8 & IGKV1-39 (n = 41)
IGHV2-5 & IGLV1-36 (n = 7)
IGHV5-51 & IGLV1-44 (n = 19)
IGHV2-70 & IGKV1-39 (n = 7)
IGHV4-4 & IGKV1-NL1 (n = 5)
IGHV1-69 & IGKV1-39 (n = 34)
IGHV1-69 & IGKV3-20 (n = 51)
IGHV1-69 & IGLV1-40 (n = 79)
IGHV3-9 & IGLV2-14 (n = 28)
IGHV3-9 & IGLV3-21 (n = 27)
IGHV3-30 & IGKV1-33 (n = 18)
IGHV3-30 & IGLV3-21 (n = 24)
IGHV3-30 & IGLV6-57 (n = 7)
IGHV4-31 & IGKV1-33 (n = 5)
RBD Position
Proportion of Antibodies with Critical Residue

Figure S2

A

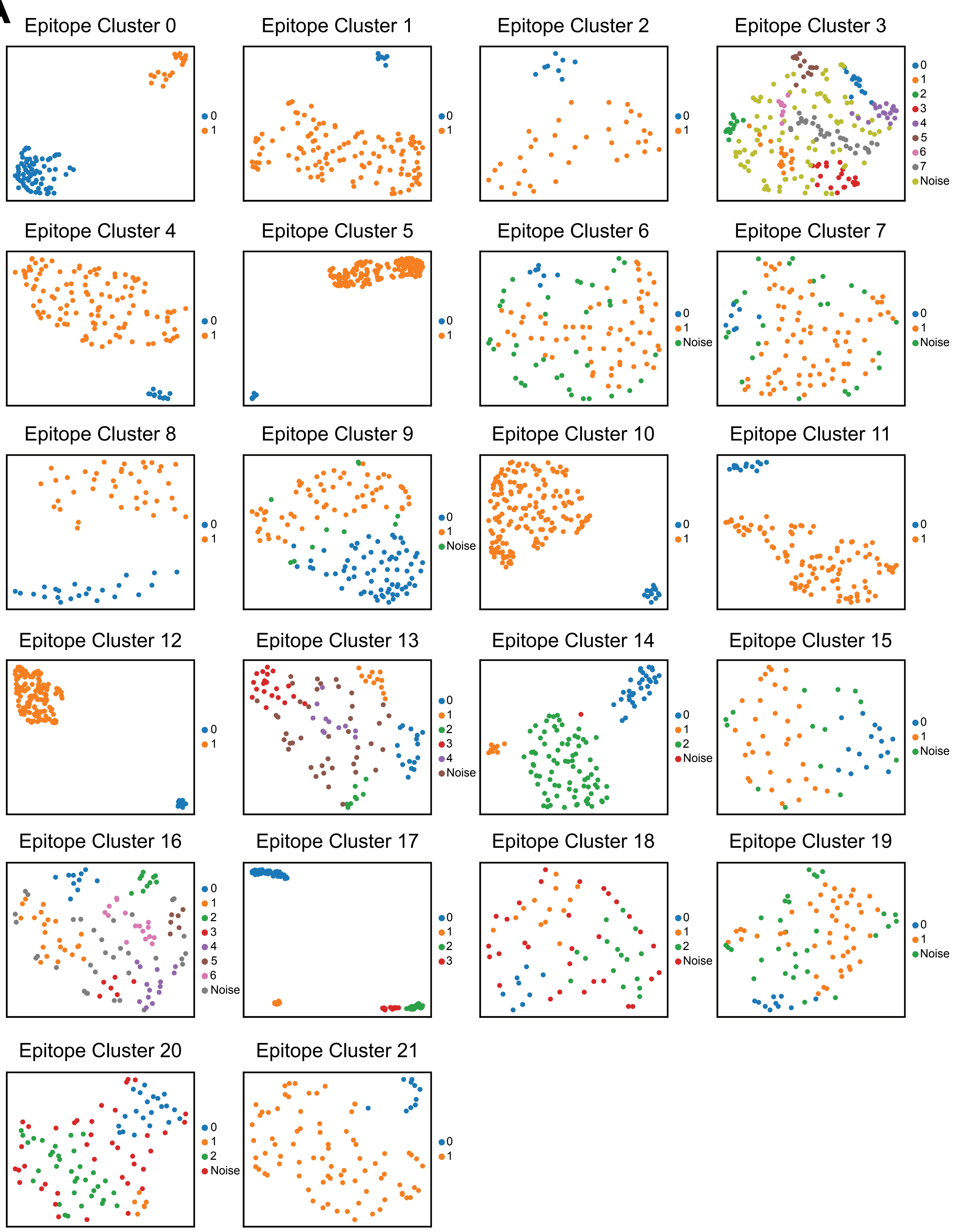

Epitope Cluster 0
Epitope Cluster 1
Epitope Cluster 2
Epitope Cluster 3
Epitope Cluster 4
Epitope Cluster 5
Epitope Cluster 6
Epitope Cluster 7
Epitope Cluster 8
Epitope Cluster 9
Epitope Cluster 10
Epitope Cluster 11
Epitope Cluster 12
Epitope Cluster 13
Epitope Cluster 14
Epitope Cluster 15
Epitope Cluster 16
Epitope Cluster 17
Epitope Cluster 18
Epitope Cluster 19
Epitope Cluster 20
Epitope Cluster 21
0
1
2
3
4
5
6
7
Noise

Figure S3

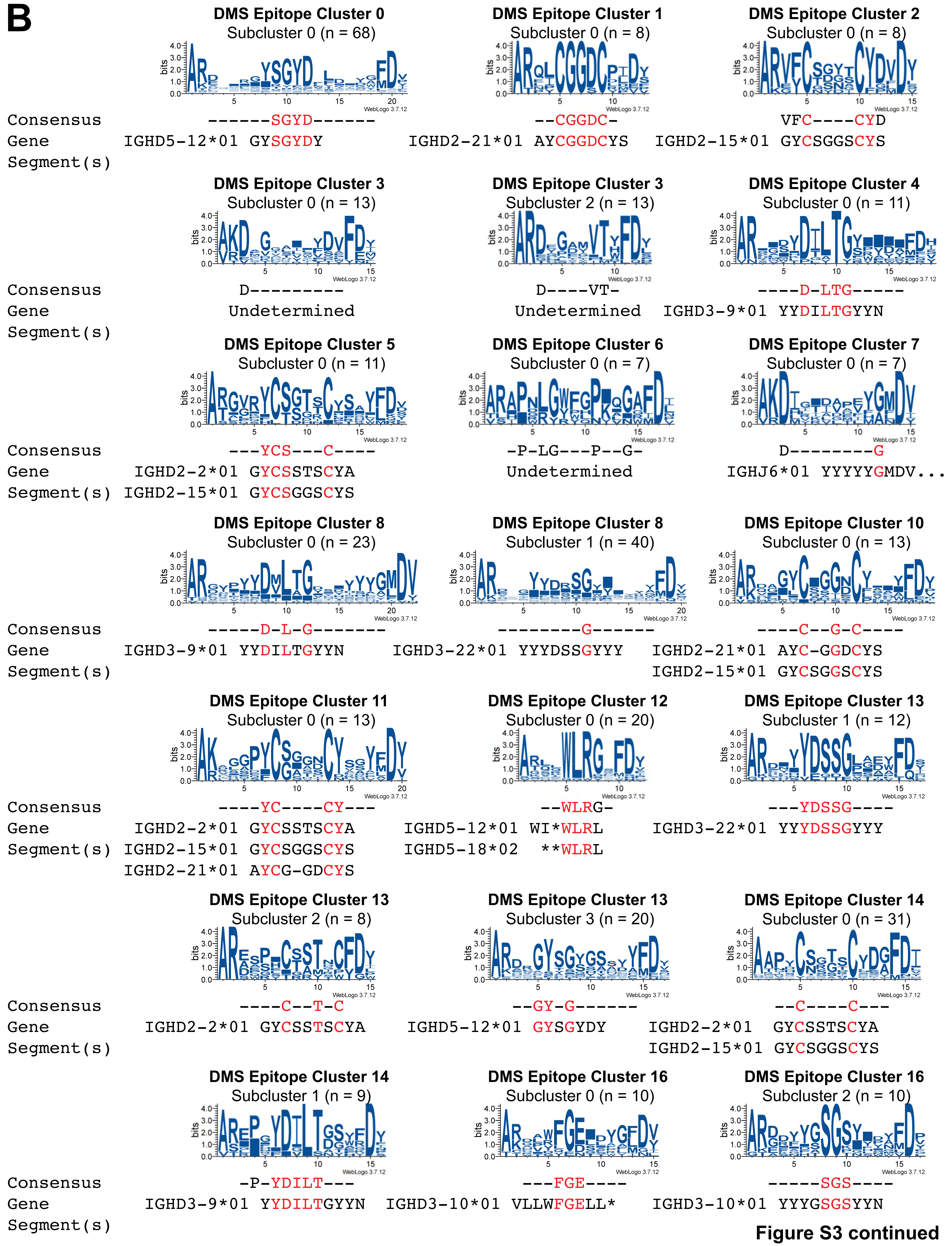

Figure S3 continued

DMS Epitope Cluster 16
Subcluster 3 (n = 6)

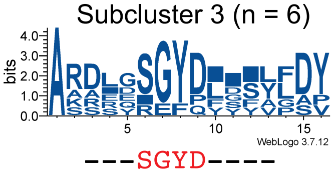

Consensus ---SGYD----
Gene Segment(s) IGHD5-12*01 GYSGYDY

DMS Epitope Cluster 16
Subcluster 5 (n = 6)

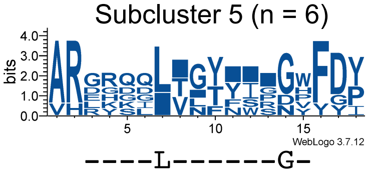

Consensus ----L------G-
Gene Segment(s) Undetermined

DMS Epitope Cluster 16
Subcluster 6 (n = 16)

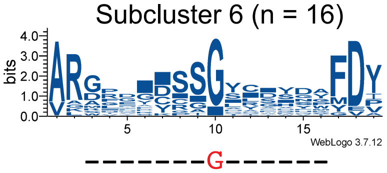

Consensus --------G------
Gene Segment(s)
IGHD3-22*01 YYYDSSGYYY
IGHD6-19*01 GYSSGWY
IGHD6-25*01 GYSSGY
IGHD2-15*01 GYCSGGSCYS

DMS Epitope Cluster 17
Subcluster 1 (n = 14)

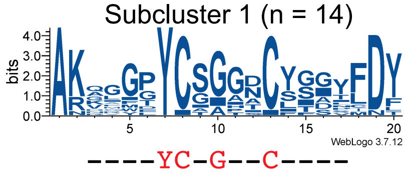

Consensus ----YC-G--C----
Gene Segment(s) IGHD2-15*01 GYCSGGSCYS

DMS Epitope Cluster 17
Subcluster 2 (n = 30)

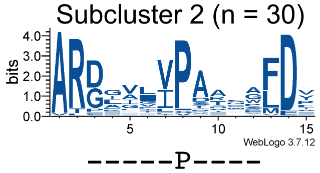

Consensus -----P----
Gene Segment(s) Undetermined

DMS Epitope Cluster 17
Subcluster 3 (n = 15)

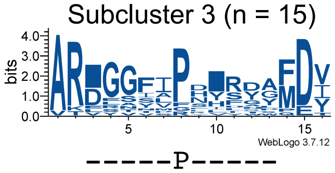

Consensus -----P-----
Gene Segment(s) Undetermined

DMS Epitope Cluster 18
Subcluster 0 (n = 9)

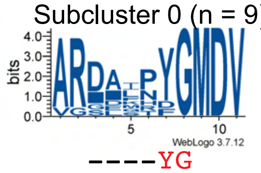

Consensus ----YG
Gene Segment(s) IGHJ6*01 YYYYYGMDV...

DMS Epitope Cluster 19
Subcluster 0 (n = 10)

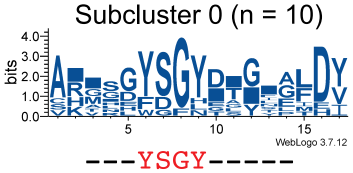

Consensus ---YSGY-----
Gene Segment(s) IGHD5-12*01 GYSGYDY

DMS Epitope Cluster 20
Subcluster 1 (n = 6)

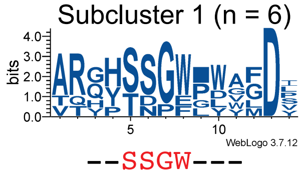

Consensus --SSGW---
Gene Segment(s) IGHD6-19*01 GYSSGWY

DMS Epitope Cluster 21
Subcluster 0 (n = 10)

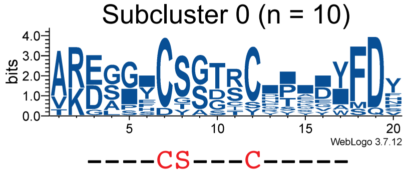

Consensus ----CS---C-----
Gene Segment(s)
IGHD2-15*01 GYCSGGSCYS
IGHD2-2*01 GYCSSTSCYA

Figure S3 continued

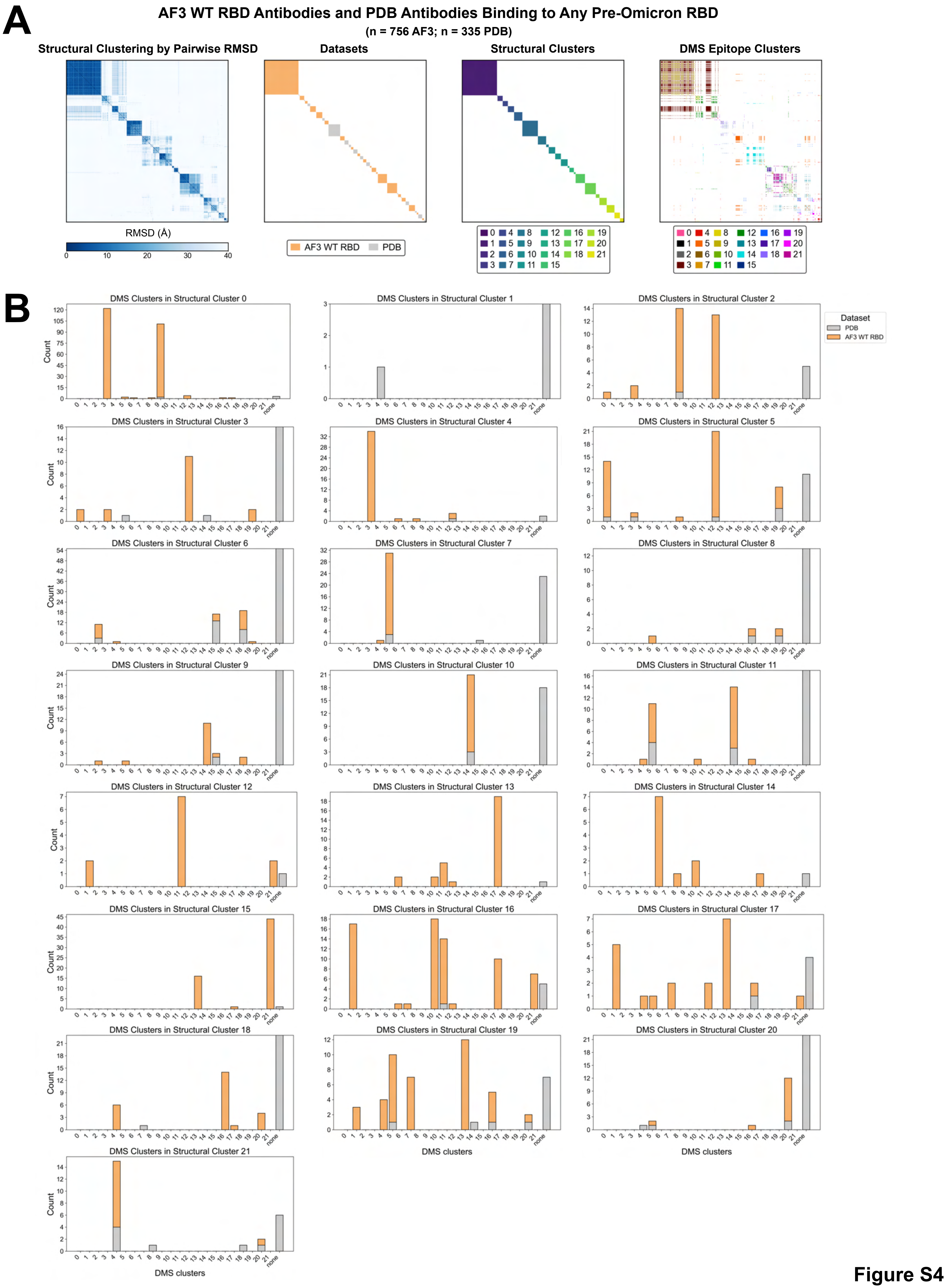
A
AF3 WT RBD Antibodies and PDB Antibodies Binding to Any Pre-Omicron RBD
(n = 756 AF3; n = 335 PDB)
Structural Clustering by Pairwise RMSD
Datasets
Structural Clusters
DMS Epitope Clusters
RMSD (Å)
AF3 WT RBD
PDB
B
DMS Clusters in Structural Cluster 0
DMS Clusters in Structural Cluster 1
DMS Clusters in Structural Cluster 2
DMS Clusters in Structural Cluster 3
DMS Clusters in Structural Cluster 4
DMS Clusters in Structural Cluster 5
DMS Clusters in Structural Cluster 6
DMS Clusters in Structural Cluster 7
DMS Clusters in Structural Cluster 8
DMS Clusters in Structural Cluster 9
DMS Clusters in Structural Cluster 10
DMS Clusters in Structural Cluster 11
DMS Clusters in Structural Cluster 12
DMS Clusters in Structural Cluster 13
DMS Clusters in Structural Cluster 14
DMS Clusters in Structural Cluster 15
DMS Clusters in Structural Cluster 16
DMS Clusters in Structural Cluster 17
DMS Clusters in Structural Cluster 18
DMS Clusters in Structural Cluster 19
DMS Clusters in Structural Cluster 20
DMS Clusters in Structural Cluster 21
Dataset
PDB
AF3 WT RBD
Count
DMS clusters
none

Figure S4

C

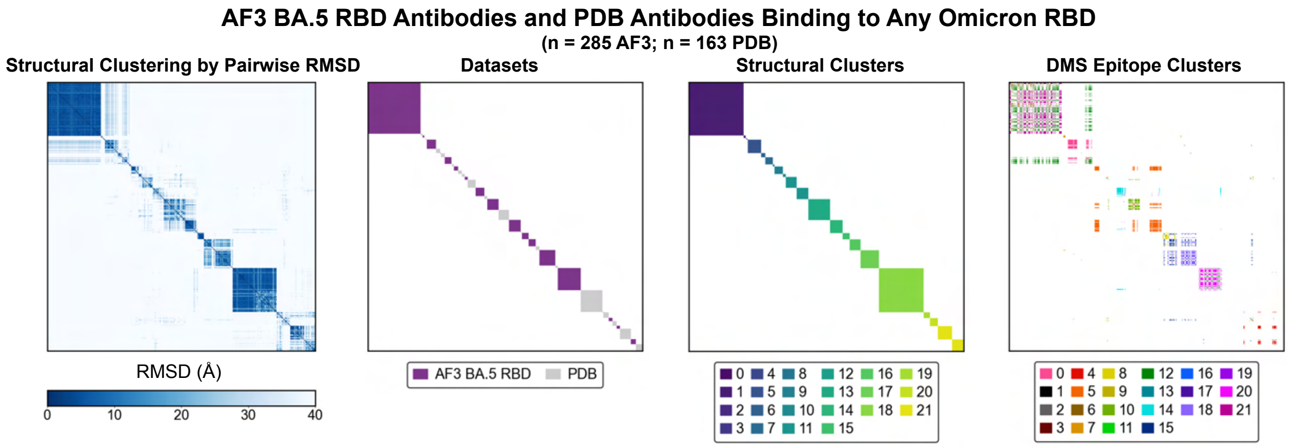

D

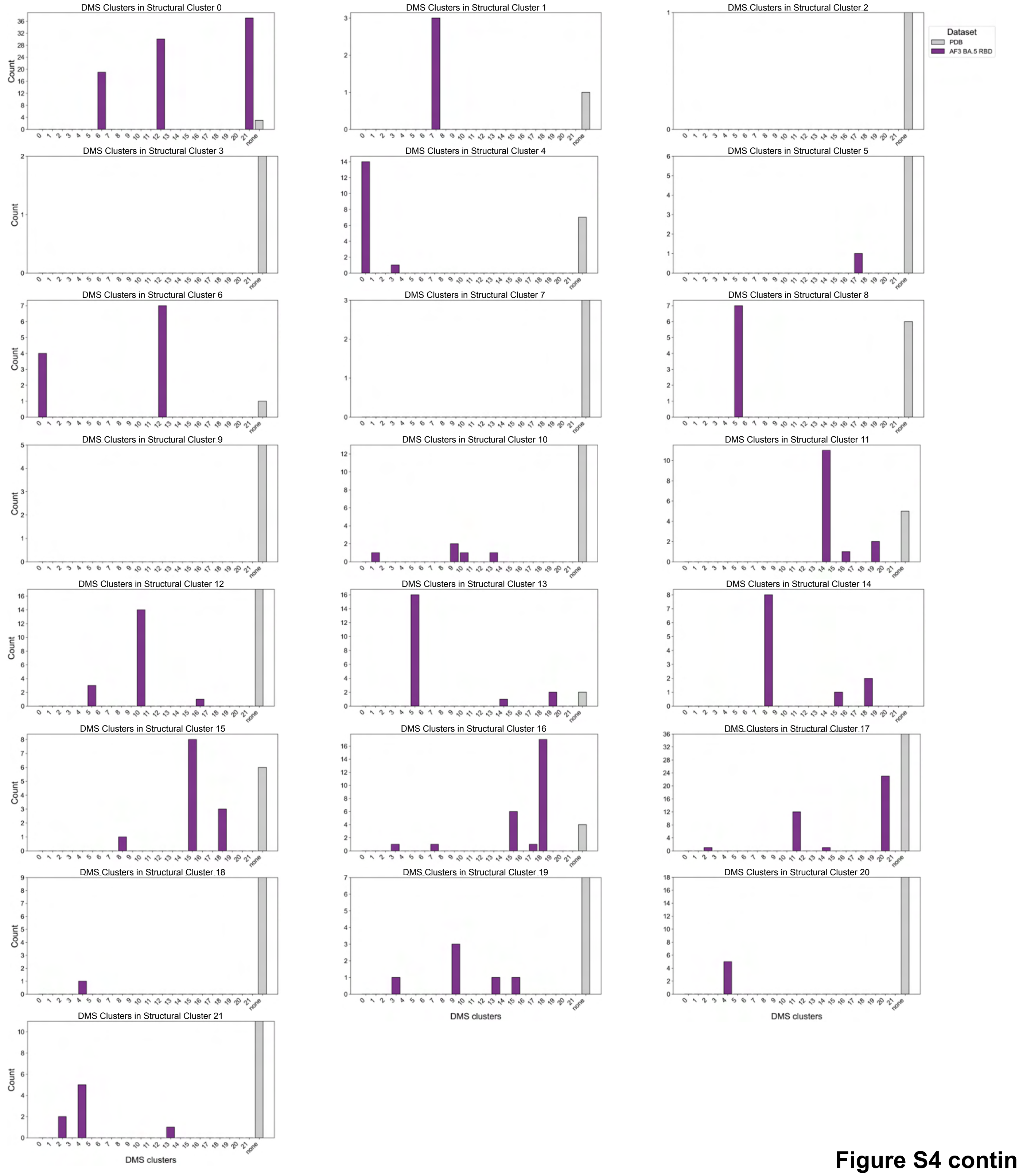

Figure S4 continued

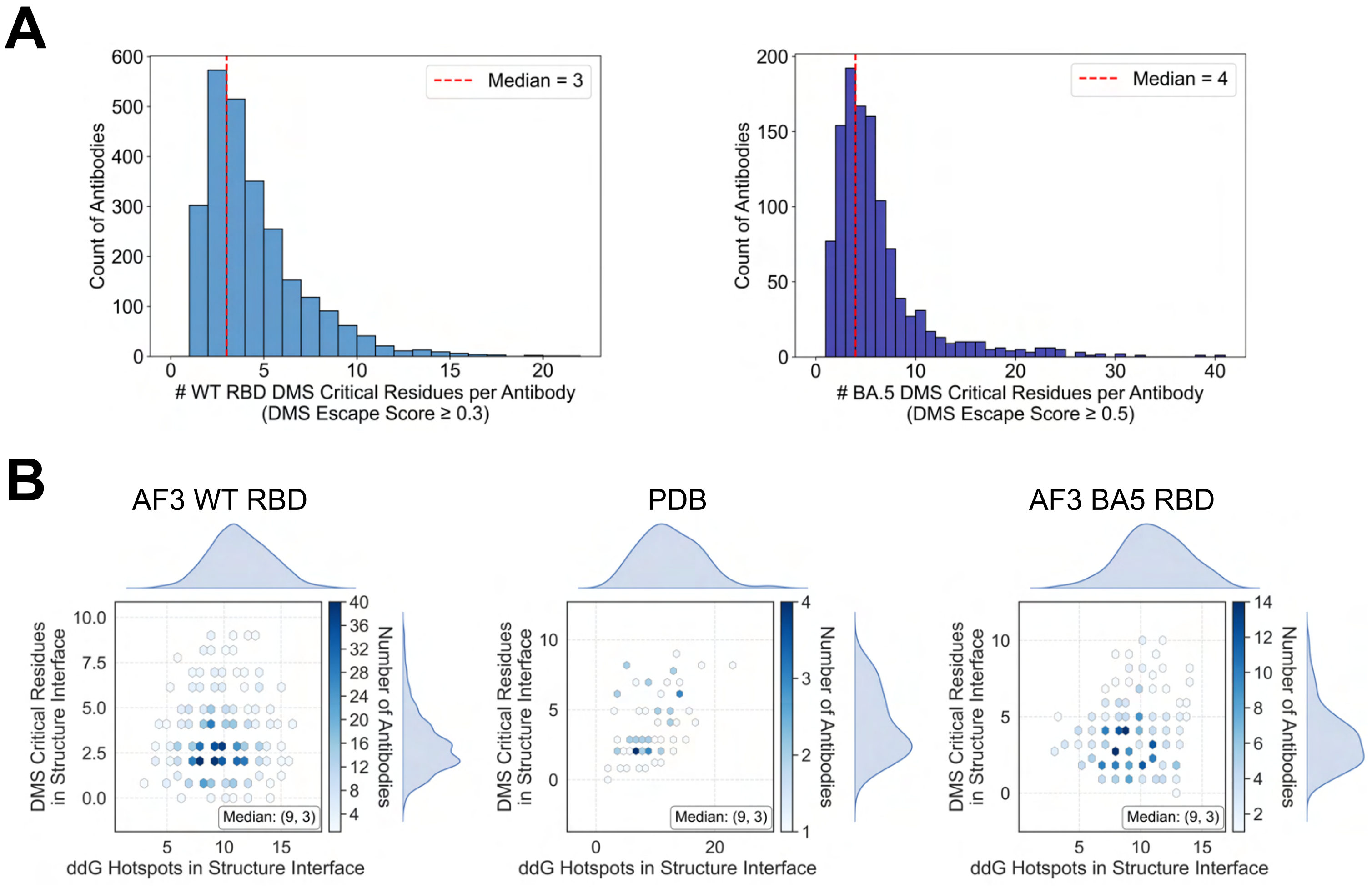
A
Median = 3
Count of Antibodies
WT RBD DMS Critical Residues per Antibody
(DMS Escape Score ≥ 0.3)
Median = 4
# BA.5 DMS Critical Residues per Antibody
(DMS Escape Score ≥ 0.5)
B
AF3 WT RBD
PDB
AF3 BA5 RBD
DMS Critical Residues in Structure Interface
ddG Hotspots in Structure Interface
Number of Antibodies
Median: (9, 3)

Figure S5

C

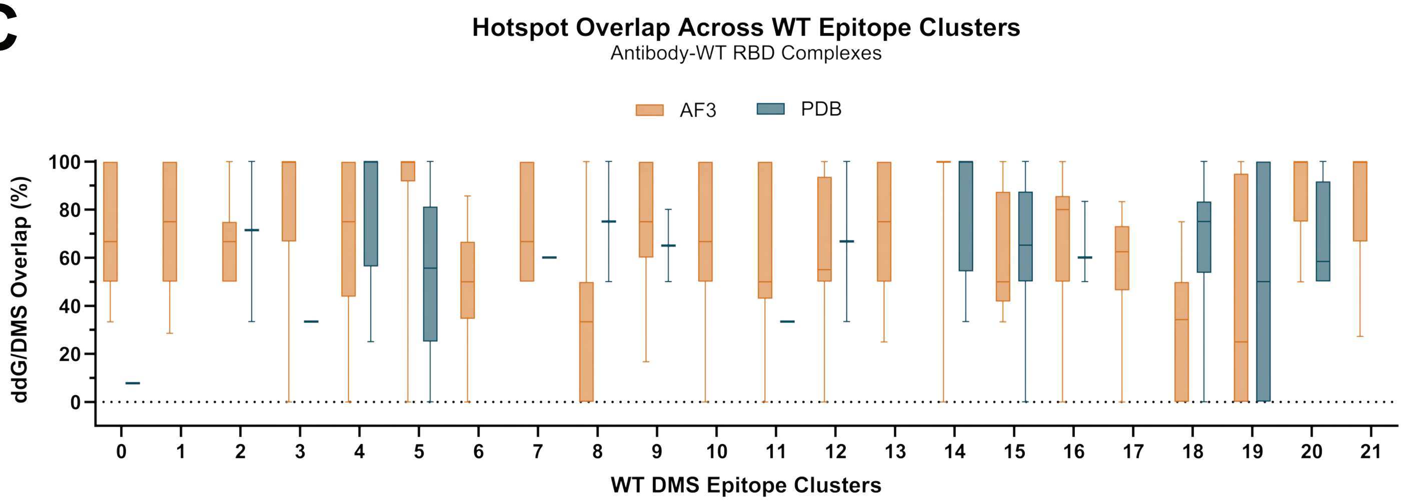

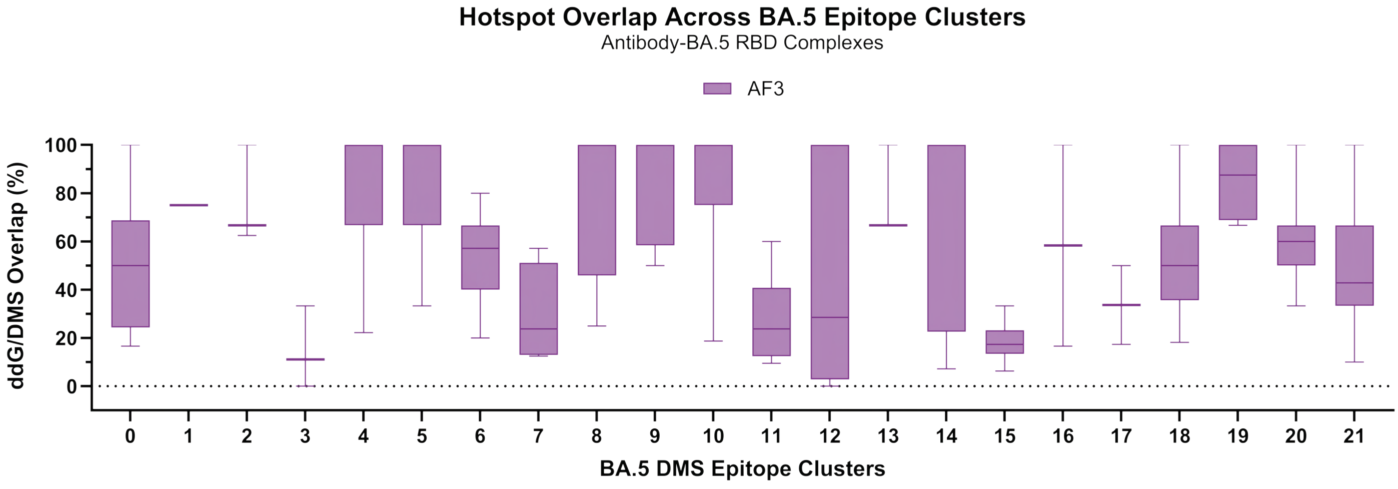

Figure S5 continued

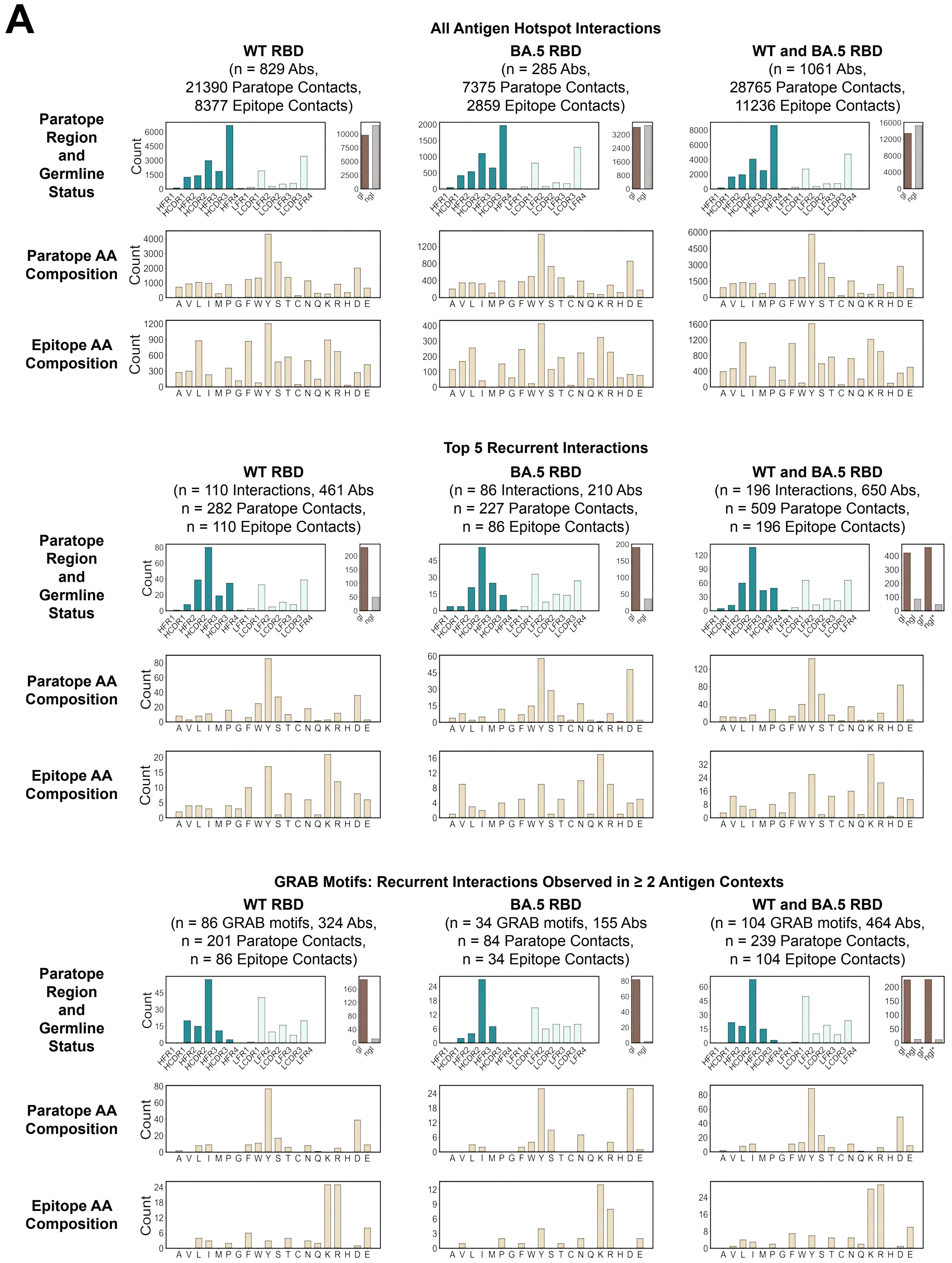
A
All Antigen Hotspot Interactions
WT RBD
(n = 829 Abs,
21390 Paratope Contacts,
8377 Epitope Contacts)
BA.5 RBD
(n = 285 Abs,
7375 Paratope Contacts,
2859 Epitope Contacts)
WT and BA.5 RBD
(n = 1061 Abs,
28765 Paratope Contacts,
11236 Epitope Contacts)
Paratope Region and Germline Status
Paratope AA Composition
Epitope AA Composition
Count
HFR1 HCDR1 HFR2 HCDR2 HFR3 HCDR3 HFR4 LFR1 LCDR1 LFR2 LCDR2 LFR3 LCDR3 LFR4
gl ngl
A V L I M P G F W Y S T C N Q K R H D E
Top 5 Recurrent Interactions
WT RBD
(n = 110 Interactions, 461 Abs
n = 282 Paratope Contacts,
n = 110 Epitope Contacts)
BA.5 RBD
(n = 86 Interactions, 210 Abs
n = 227 Paratope Contacts,
n = 86 Epitope Contacts)
WT and BA.5 RBD
(n = 196 Interactions, 650 Abs,
n = 509 Paratope Contacts,
n = 196 Epitope Contacts)
gl ngl gl* ngl*
GRAB Motifs: Recurrent Interactions Observed in ≥ 2 Antigen Contexts
WT RBD
(n = 86 GRAB motifs, 324 Abs,
n = 201 Paratope Contacts,
n = 86 Epitope Contacts)
BA.5 RBD
(n = 34 GRAB motifs, 155 Abs
n = 84 Paratope Contacts,
n = 34 Epitope Contacts)
WT and BA.5 RBD
(n = 104 GRAB motifs, 464 Abs,
n = 239 Paratope Contacts,
n = 104 Epitope Contacts)

Figure S6

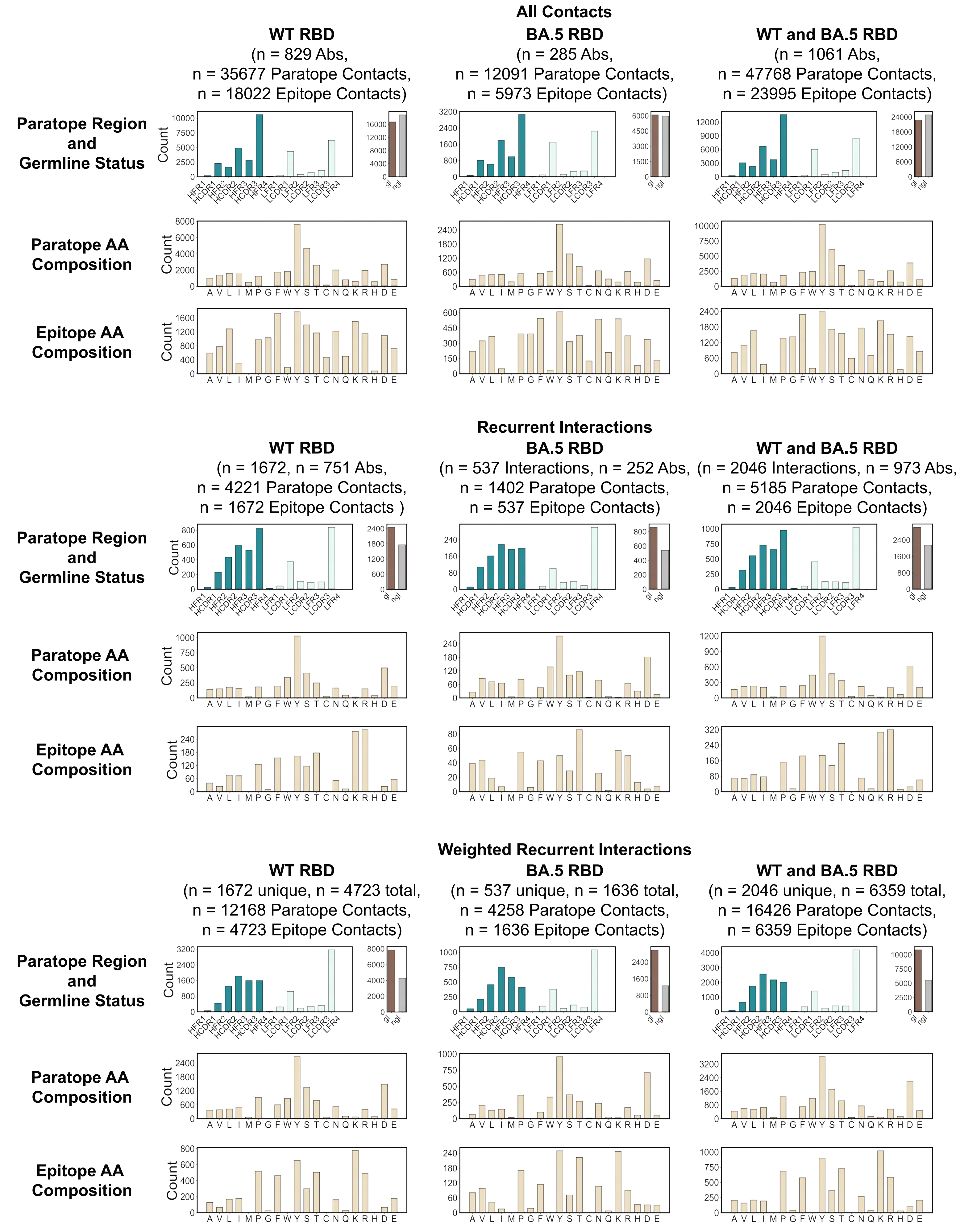

Figure S6 continued

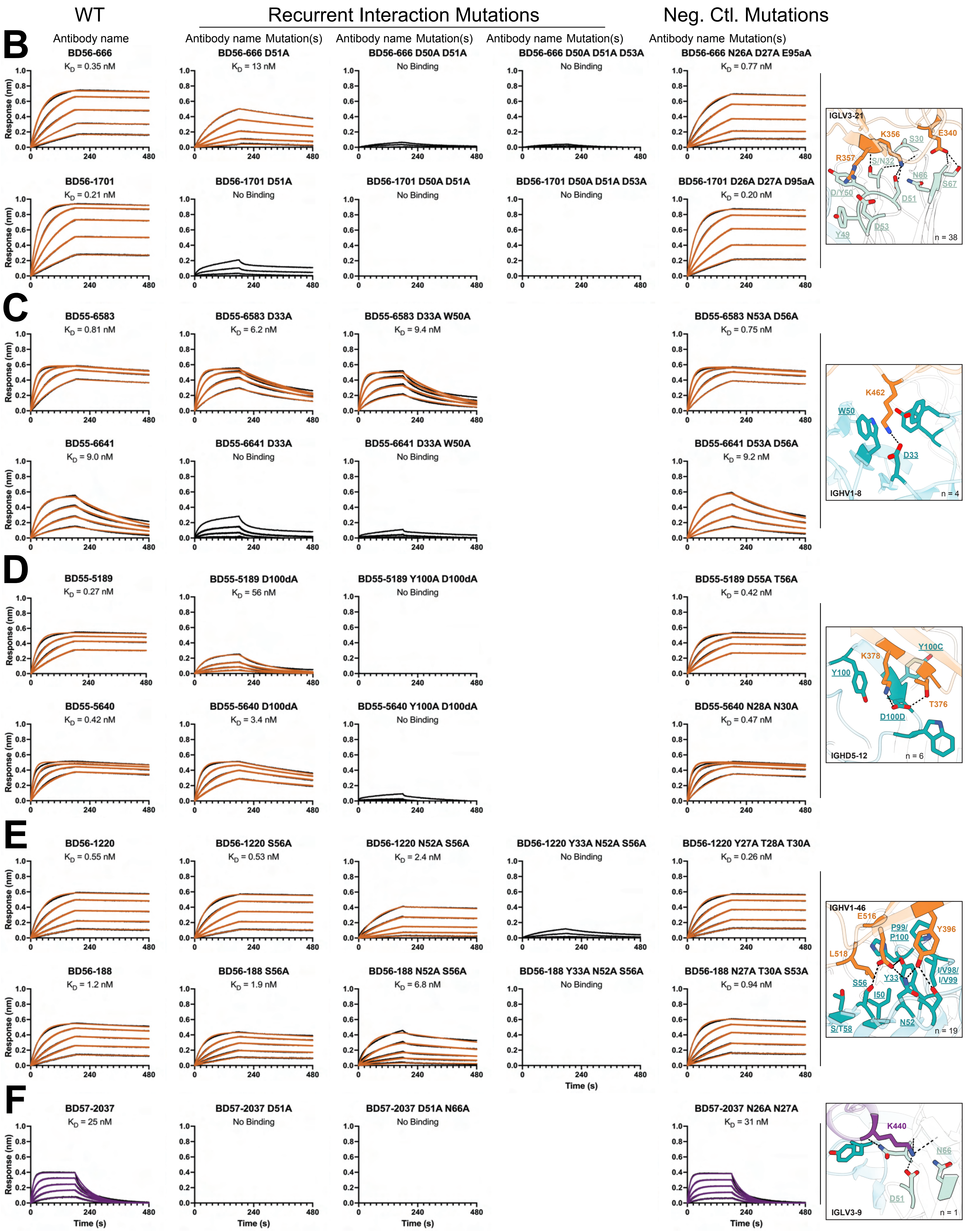

Figure S6 continued

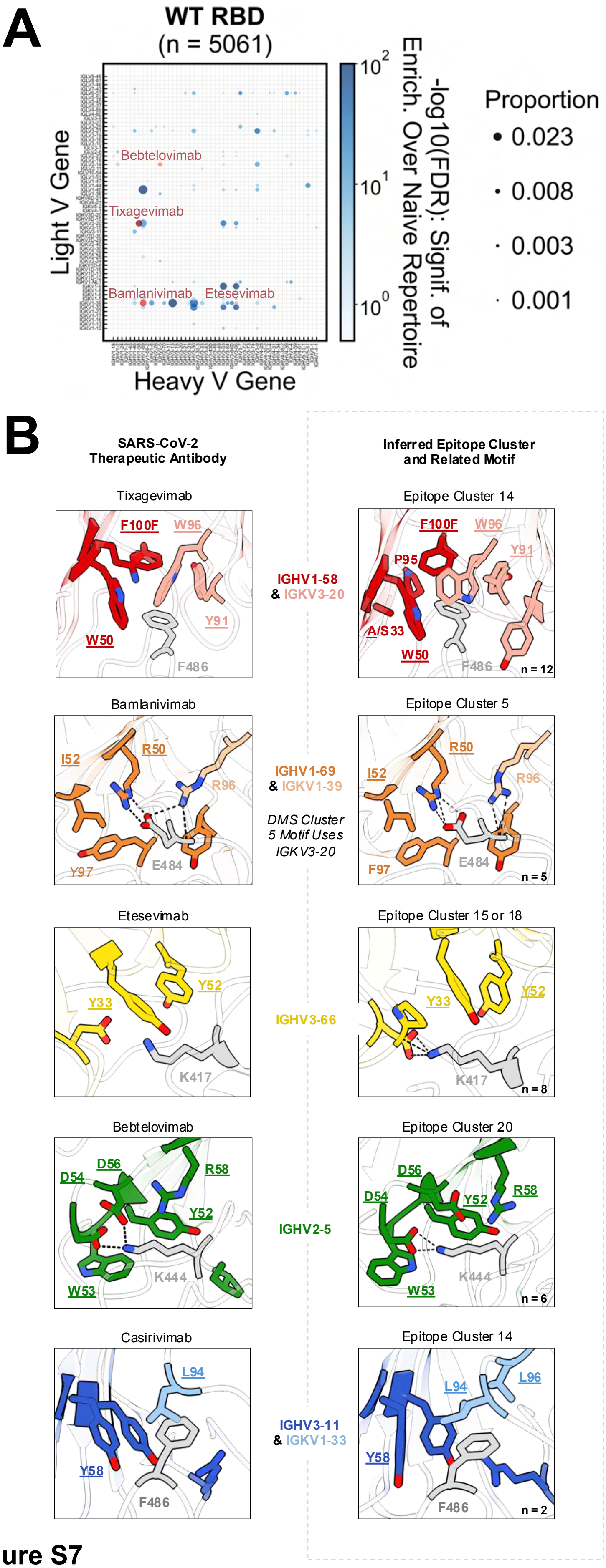
A
WT RBD
(n = 5061)
Light V Gene
Heavy V Gene
Bebtelovimab
Tixagevimab
Bamlanivimab
Etesevimab
-log10(FDR): Signif. of Enrich. Over Naive Repertoire
Proportion
0.023
0.008
0.003
0.001
B
SARS-CoV-2 Therapeutic Antibody
Inferred Epitope Cluster and Related Motif
Tixagevimab
Epitope Cluster 14
F100F
W96
Y91
W50
F486
P95
A/S33
n = 12
IGHV1-58 & IGKV3-20
Bamlanivimab
Epitope Cluster 5
I52
R50
R96
Y97
E484
F97
n = 5
IGHV1-69 & IGKV1-39
DMS Cluster 5 Motif Uses IGKV3-20
Etesevimab
Epitope Cluster 15 or 18
Y33
Y52
K417
n = 8
IGHV3-66
Bebtelovimab
Epitope Cluster 20
D54
D56
R58
Y52
W53
K444
n = 6
IGHV2-5
Casirivimab
Epitope Cluster 14
L94
L96
Y58
F486
n = 2
IGHV3-11 & IGKV1-33

Figure S7

C

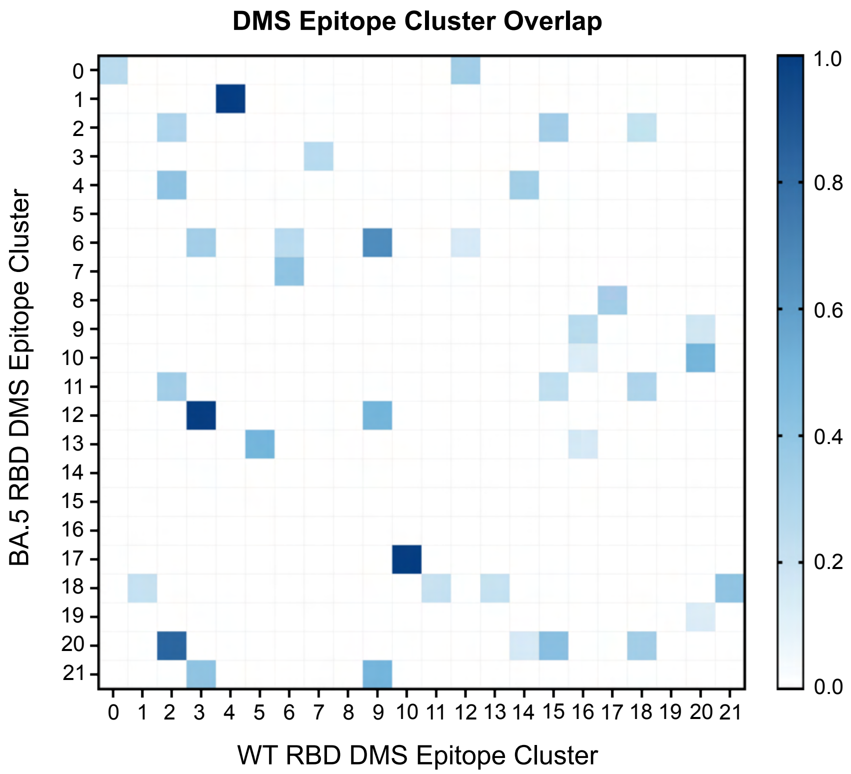

WT RBD
DMS Epitope Clusters

Clust.	Critical Residue(s)
0	376, 378, 408
1	357, 468
2	455, 456, 475, 487, 489
3	383, 386
4	346
5	484, 490
6	391, 393, 518, 525
7	348, 352
8	381, 413
9	383, 384, 385, 386
10	462
11	357, 396
12	378, 384
13	356, 468
14	486, 487
15	417, 455, 456, 475
16	444, 447, 448, 449, 450, 452, 490
17	396, 514, 516
18	456, 475
19	503, 504
20	444, 445, 446, 447
21	356, 357, 468

BA.5 RBD
DMS Epitope Clusters

Clust.	Critical Residue(s)
0	377, 378
1	346
2	403, 455, 456, 505
3	336, 348, 361
4	487, 489
5	439, 440
6	383, 384, 385, 386, 391, 525
7	391, 524, 525
8	516
9	447, 449, 498
10	444, 445
11	420, 421, 456, 473, 475, 480, 489
12	383, 386
13	490
14	505
15	336, 353, 361, 399, 454, 466, 495, 497
16	498
17	462
18	336, 356, 357, 361
19	439, 445, 498, 499, 500
20	455, 456, 460, 475, 487, 489
21	336, 361, 383, 385, 386

Figure S7 continued

D

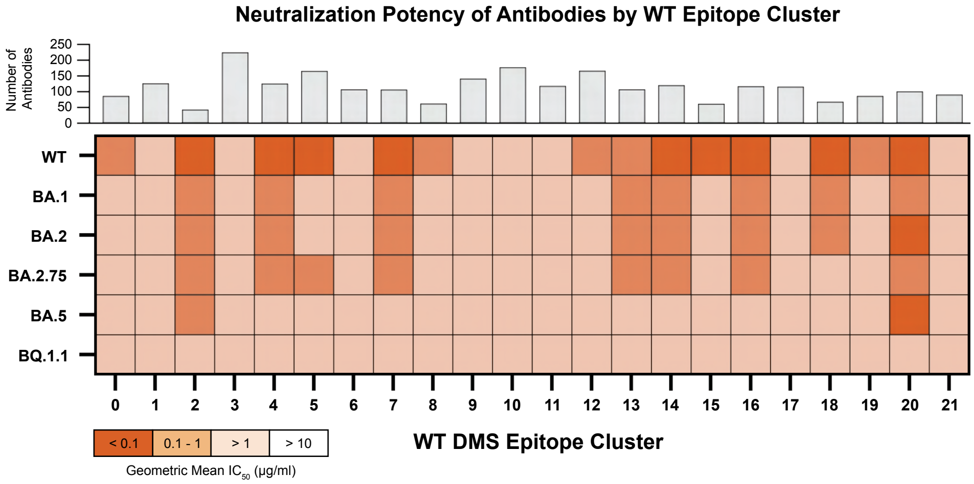

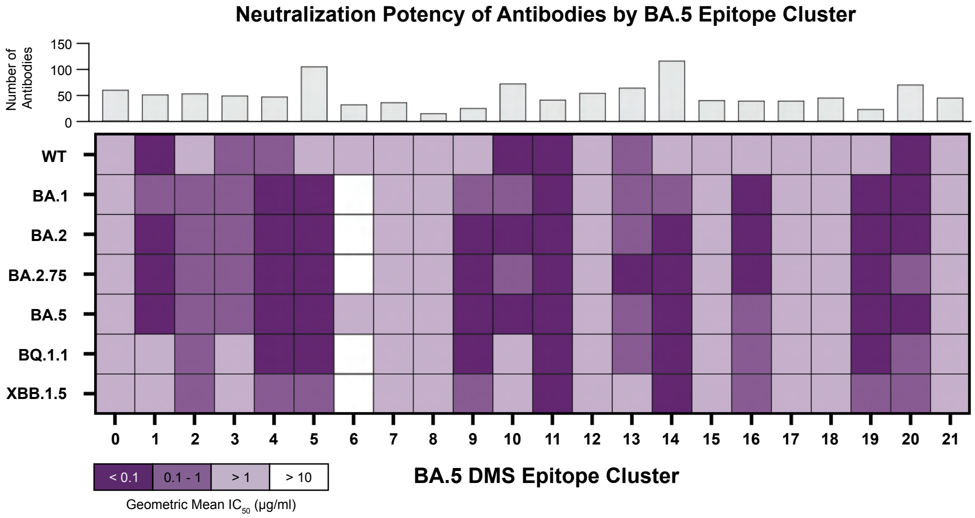

Figure S7 continued
