# Supplementary material for: Systems-Scale Structural Modeling Reveals the Germline Architecture of Immunodominance
Source: bioRxiv. 2026 Jun 4:2026.06.02.729678. Preprint. [Version 1] doi: 10.64898/2026.06.02.729678 (PMC13252370; doi:10.64898/2026.06.02.729678)
Supplement: 2 [file NIHPP2026.06.02.729678v1-supplement-2.pdf]

bioRxiv preprint doi: https://doi.org/10.64898/2026.06.02.729678; this version posted June 4, 2026. The copyright holder for this preprint (which was not certified by peer review) is the author/funder, who has granted bioRxiv a license to display the preprint in perpetuity. It is made available under a CC-BY 4.0 International license.

Supplemental information titles and legends

Supplementary Discussion

Point #1

Antibodies in different DMS epitope clusters exhibit varying patterns of neutralization potency (**Figure S7D**).

Point #2

Many of the HCDR3 motif clusters also featured biased V gene segment usage (**Supplementary Data**).

Upon generation of the AF3 predicted dataset, we examined whether residues within conserved HCDR3 motifs generally contacted DMS-critical residues or ddG hotspots on the RBD, which could account for their conservation. In almost all cases, they did (**Supplementary Data**).

Point #3

We note that none of IgLV3-10, IgLV3-25, IgLV6-57, IgLV3-1, or IgLV5-37, i.e. genes that harbor lysine-specific GRAB motifs similar to that in IgLV3-21, share the S/N32, Y49, D/Y50, and D53 residues predicted to contact RBD R357. In particular, IgLV3-10, IgLV3-25, and IgLV3-1 have positively charged residues in one of these positions that could repel RBD R357. (Germline-encoded residues at these positions are as follows:

bioRxiv preprint doi: https://doi.org/10.64898/2026.06.02.729678; this version posted June 4, 2026. The copyright holder for this preprint (which was not certified by peer review) is the author/funder, who has granted bioRxiv a license to display the preprint in perpetuity. It is made available under a CC-BY 4.0 International license.

IgLV3-10: Y32, Y49, E50, K53; IgLV3-25: Y32, Y49, K50, E53; IgLV3-1: Y32, Y49, Q50, K53; IgLV6-57: Y32, Y49, E50, Q53; IgLV5-37: N32, Y49, Y50, and G53[34–36].)

Point #4

One specific residue, N32 in IgLV3-21, was not annotated as germline-encoded. However, since the S-to-N substitution requires multiple nucleotide changes and is therefore less likely to arise through somatic hypermutation, it is plausible that this N32 is germline-encoded in an as-yet-undescribed IgLV3-21 allele, potentially enriched in the Chinese population from which the dataset was derived.
